# Effect of Body Position and Support Surface on the Postural Control Challenge During the Pallof Press Exercise: A Smartphone Accelerometer-Based Study

**DOI:** 10.3390/medicina61020312

**Published:** 2025-02-11

**Authors:** Casto Juan-Recio, Amaya Prat-Luri, Heidy Rondón-Espinosa, David Barbado, Francisco J. Vera-Garcia

**Affiliations:** 1 Sports Research Centre, Department of Sport Sciences, Miguel Hernández University of Elche, Avda. de la Universidad s/n, 03202 Alicante, Spain; cjuan@umh.es (C.J.-R.); hrondon@unal.edu.co (H.R.-E.); dbarbado@umh.es (D.B.); fvera@umh.es (F.J.V.-G.); 2 Institute for Health and Biomedical Research, ISABIAL Foundation, Miguel Hernández University of Elche, Avda Pintor Baeza, 12—Planta 5a Centro de Diagnóstico, 03010 Alicante, Spain

**Keywords:** core stabilization, training intensity, load progression, field testing, posturography, trunk control

## Abstract

*Background and objectives*: Although different variations of the Pallof press exercise are commonly performed in sports and fitness settings to increase core stability, the intensity/difficulty of these variations is unknown and therefore it is difficult to control the training load and establish exercise progressions. This study aimed to compare and rank the postural control challenge imposed by five different isometric variations of the Pallof press exercise through a smartphone accelerometer placed on the participants’ pelvis and to explore sex differences in the lumbopelvic postural control during the exercise performance. *Materials and Methods*: Twelve physically active participants completed two testing sessions in which they performed two sets of five different isometric variations of the Pallof press exercise (changing the body position and/or the support surface: kneeling on a foam pad, feet together standing on the floor, tandem stance on the floor, feet together standing on a hemisphere ball, and tandem stance on a hemisphere ball). After confirming the acceleration data reliability (intraclass correlation coefficients ≥ 0.72 and typical errors ≤ 17%), a repeated measure ANOVA was carried out to classify the Pallof press variations according to the postural control challenge imposed on the participants and to analyze sex differences on postural control. *Results*: Significant effects were found for the within-subject factor exercise variations but not for the between-subject factor sex. Pairwise comparisons showed that the exercise variations performed on the hemisphere ball (feet together standing: 0.55 m/s^2^; tandem stance: 0.61 m/s^2^) imposed higher postural control demands than those performed on the other surfaces (kneeling on a foam pad: 0.17 m/s^2^; feet together standing on the floor: 0.22 m/s^2^; tandem stance on the floor: 0.31 m/s^2^). In addition, the tandem stance on the floor produced higher lumbopelvic accelerations than the Pallof press kneeling variation. *Conclusions*: The Pallof press performance in standing rather than kneeling (i.e., reducing the base of support and raising the center of gravity and the height of the lateral force applied by the elastic band) and on a hemisphere ball increased the exercise difficulty compared to more stable surfaces. This information could help to modulate the difficulty and establish progressions for this exercise in physically active young males and females.

## 1. Introduction

Nowadays, exercise programs aimed at improving core stability (CS) are common elements in fitness, physical education, rehabilitation, and amateur and professional sports [1,2,3,4,5]. However, despite its popularity, there is no clear evidence of the benefits of CS in different contexts, especially in sports performance [4,6,7]. The lack of specificity of both the exercises and the tests used in the experimental studies could be highlighted among the different reasons why CS exercises might not have shown benefits for athletes [8]. In this sense, according to the study of Barbado et al. [8], only those tests that require sport-specific trunk stability demands are suitable for measuring this quality in athletes practicing that sport. Similarly, trunk stability exercises performed by these athletes should challenge the stability in similar conditions to those specific to their sport [9,10], which in many cases are far from conventional training.

Generally, conventional CS exercises are performed in quadruped, prone, supine, or lateral decubitus positions [11,12,13,14]. This type of workout challenges postural control, inducing moderate trunk muscle activations [15,16,17,18,19,20] without exposing the spine to high levels of stress [21]. In addition, they do not need expensive equipment and they are relatively easy for health and sports professionals to monitor. However, this type of workout is not very ecological according to the usual stability needs of sports skills nor for coping with the demands of daily living tasks, which usually happen in more vertical positions (standing, sitting, etc.). In this sense, conventional floor-based CS exercises could be more useful for beginners than for athletes or people with high postural control, who require more ecological training that uses CS exercises performed in specific positions.

The Pallof press [22,23,24,25,26], is a transverse plane or trunk rotation exercise that basically consists of keeping the spine and pelvis in a neutral position against the torsion moment generated by a lateral force applied on the trunk through a cable or a rubber/elastic band held by the participant, could be highlighted between the CS exercises performed in standing positions. During the conventional form of this exercise, the participant tries to keep the trunk still while extending and flexing the elbows (against the lateral force), which modifies the lever arm and therefore the torsional moment on the trunk [22,23]. There are different variations of the Pallof press technique depending on the body position (standing, seated, half-kneeling, etc.), whether the cable/band is held with one or two hands, if the exercise is performed with or without limb movements, etc. [22,23,24]. However, although a Pallof press progression for the design of training programs has been proposed by Mullane et al. [22], there is a lack of objective criteria to assess the intensity of different variations of this exercise and ultimately establish difficult progressions are lacking.

The main objective of this study was to develop a progression of five isometric variations of the Pallof press exercise performed in different body positions (kneeling, feet together standing, and tandem stance) and on different support surfaces (a foam pad, the floor, and a hemisphere ball). Considering the reliability, low cost, and ease of use of the accelerometers embedded in smartphones to establish CS exercise progressions based on the postural control challenge imposed on the participants by these exercises [11,12,27,28], this posturographic technique was used to assess the difficulty the participants had to perform each Pallof press variation. In addition, as an exploratory aim, lumbopelvic postural control comparing females and males was also performed to provide preliminary data for the calculation of sample size in future studies that target sex differences. Finally, this study explored the acceleration data consistency, since we have no evidence that the smartphone accelerometry has been used before during the execution of this exercise.

## 2. Method

### 2.1. Participants

A total of 6 males (age: 30.5 ± 11.0 years; height: 173 ± 0.7 cm; body mass: 77.0 ± 6.7 kg) and 6 females (age: 25.6 ± 3.9 years; height: 165.9 ± 5.7 cm; body mass: 61.9 ± 6.0 kg) participated in this study. The participants were physically active and performed at least 150 min of moderate physical activity or 75 min of vigorous physical activity per week (resistance exercises, running, cycling, gymnastics, etc.). They did not present any history of hip, spinal, shoulder, or abdominal surgery, neurological disorders, inguinal hernia, or episodes of low back pain, which required medical treatment in the last 6 months and they had not participated in any structured and specific core training program in the 6 months prior to this study. The researchers informed of the risks of this study to the participants and they filled out a written informed consent according to the Declaration of Helsinki and approved by the Miguel Hernández University Office of Research Ethics (DCD.CJR.230630). Sample size estimation was based on a conservative estimation of the ANOVA effect size *f* calculated from the results of a previous study on the progressions of CS exercises [11]. Specifically, the sample size was estimated in G power 3.1.9.2 version with an effect size f = 0.40, α = 0.05, and power = 80%. The sample size calculated was 10 and we added 20% for possible dropouts, resulting in 12 participants.

### 2.2. Procedures

The participants completed two testing sessions (of approximately 45–60 min each separated one week apart) in which they performed two sets of five variations of the Pallof press after a 10-min standardized warming up [28] in each session (1 min rest between variations and 5 min rest between sets). They performed both sessions in the same laboratory controlling some environmental factors such as temperature, humidity, type of surface, etc. The participants maintained the required posture for 15 s and the average acceleration (m/s^2^) of the lumbopelvic area was registered using the Coremaker application. For this purpose, an accelerometer integrated into a smartphone (iPhone SE model, MHGQ3QL/A; USA) was placed over the sacrum using an elastic belt. They had two opportunities to complete each variation (if not, it was concluded that they were unable to perform it). The researchers asked the participants not to engage in any vigorous physical activity for at least 24 h before each test day.

### 2.3. Pallof Press Variations

**Figure 1:**
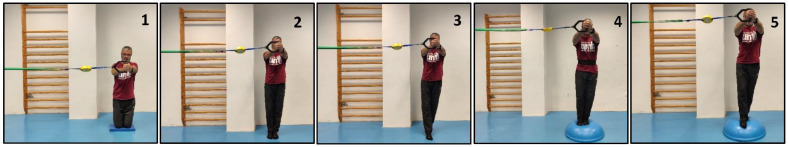
Images of a participant performing the Pallof press variations: (1) kneeling on a foam mat; (2) feet together standing on the floor; (3) tandem standing on the floor; (4) feet together standing on a hemisphere ball; (5) tandem standing on a hemisphere ball.

**Figure 2:**
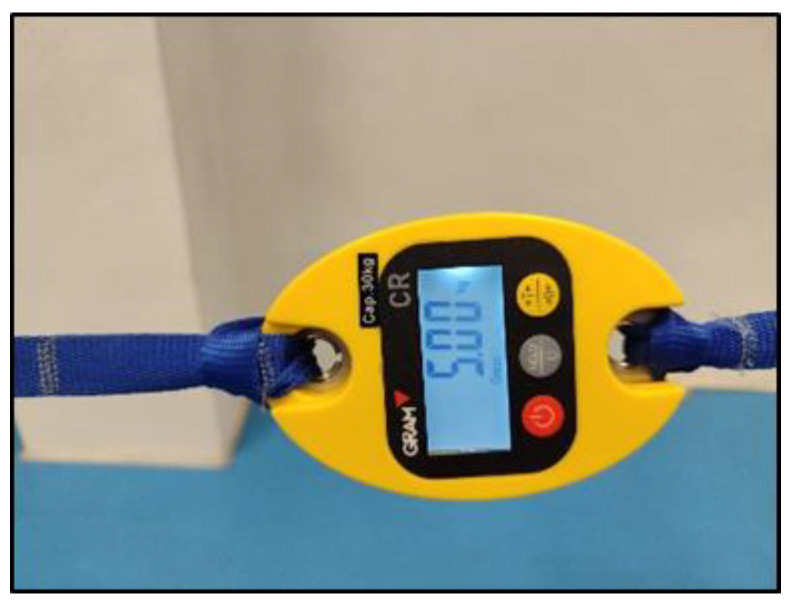
Image of the dynamometer showing 5 kg of lateral force generated by the elastic band on a participant.

The participants performed five isometric Pallof press variations based on different mechanical criteria to increase difficulty/intensity in postural control: (i) reducing the base of support; (ii) raising the center of gravity and the height of the lateral force applied by the elastic band; (iii) using unstable surfaces, and following the same order as shown in Figure 1: (1) kneeling on a semi-rigid foam pad (30 × 10 × 5 cm; 80 kg/m^3^); (2) with their feet together standing on the floor; (3) tandem stance on the floor; (4) with their feet together standing on a hemisphere ball (turtle model T2; dimensions of 65 cm diameter by 25 cm height); and (5) tandem stance on a hemisphere ball. Although the Pallof press is usually performed with upper limb movement, in this study, it was carried out with the elbows extended to avoid sudden trunk movements and to improve the reliability of the accelerometry data. During the execution of each Pallof press variation, the participant, keeping the arms extended perpendicular to the body at shoulder height, used both hands to hold a handle attached to an elastic band anchored to a pulley machine which facilitated the placement of the elastic band on a horizontal plane (i.e., the elastic band direction had to follow a straight trajectory with respect to the position of the hands). Finally, a digital dynamometer (Gram Lite CR-30; Spain) attached to the elastic band (Figure 2) was used to standardize the resistance of the elastic band according to the participant’s body weight. A pilot study was carried out to establish the lateral force generated by the elastic band on the participants according to their body weight. The main results are as follows (body weight: lateral force): 50–60 kg: 4 kg; 60–70 kg: 4.5 kg; 70–80 kg: 5 kg; 80–90 kg: 5.5 kg; >90 kg: 6 kg.

### 2.4. Statistical Analysis

Descriptive statistics, including mean (average of the two trials of each session) and standard deviations, were calculated for the lumbopelvic acceleration of each exercise variation of the entire sample. The normal distribution of the lumbopelvic acceleration data, was checked using a Kolmogorov-Smirnov test with the Lilliefors correction (*p* < 0.05). The researchers used a spreadsheet designed by Hopkins [29] to analyze test-retest reliability through the intraclass correlation coefficient (ICC_3,1_) with its confidence limits set at 95% and the typical error (standard deviation of the difference between the two sessions divided by √2) [30]. The following criteria were used to interpret the ICC values: excellent (0.90–1.00), good (0.70–0.89), fair (0.50–0.69), and low (<0.50) [31]. A typical error ≤ 20% was considered acceptable for the posturographic analysis based on previous reliability data for the procedures involved [28].

A 2-way mixed analysis of variance (ANOVA) was performed to classify the Pallof press variations according to the postural control challenge imposed on the participant and to analyze sex differences with the data obtained in the second session. The within-subject factor was exercise variations (five Pallof press variations), whereas the between-subject factor was sex (males and females). Student *t* analyses with Bonferroni correction were carried out for pairwise comparisons. Cohen’s *f* was used as a measure of effect size for ANOVA and was interpreted as follows: small, 0.10; medium, 0.25, and large, 0.40. Standardized mean differences (Cohen’s *d*) were used to quantify as effect size index for pairwise comparisons, being interpreted as follows: small, 0.20; medium, 0.50, and large, 0.80 [25]. All the statistical analyses were performed with the JASP package (version 0.18.1, Netherlands), establishing significance at *p* < 0.05.

## 3. Results

Table 1 shows the descriptive statistics for session 1 and session 2 in which mean lumbopelvic acceleration values ranged from 0.17 to 0.61 m/s^2^. Regarding data consistency, the relative reliability was good with ICC ≥ 0.72, and the absolute reliability was acceptable, according to previous studies [2], with typical errors ≤ 17%. There were no statistical differences between sessions 1 and 2 for the mean acceleration values obtained from each Pallof press variation.

**Figure 3:**
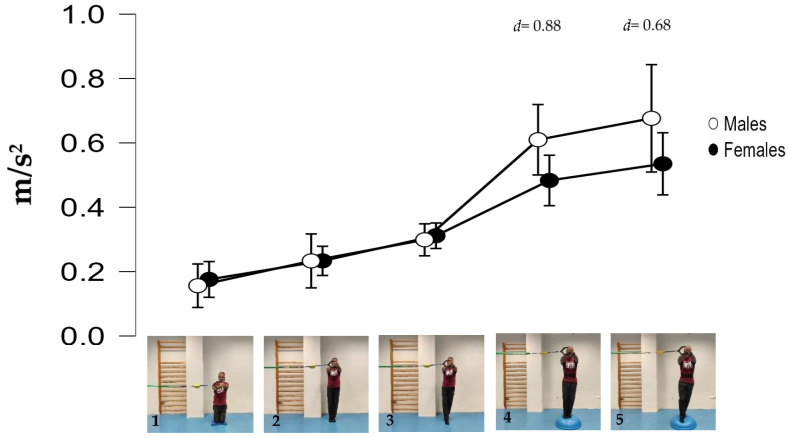
Mean lumbopelvic accelerations of each variation by sex: (1) kneeling on a foam mat; (2) feet together standing on the floor; (3) tandem standing on the floor; (4) feet together standing on a hemisphere ball; (5) tandem standing on a hemisphere ball. *D =* Cohen’s *d* statistic (standardized mean differences between males and females).

**Figure 4:**
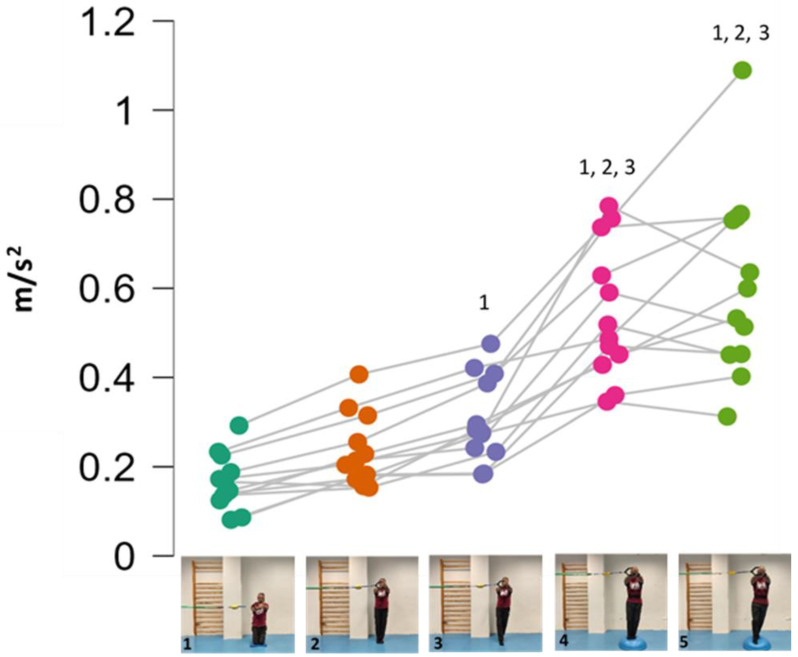
Progression of Pallof press variations ranked by the level of postural control challenge (i.e., lumbopelvic acceleration): (1) kneeling on a foam mat; (2) feet together standing on the floor; (3) tandem standing on the floor; (4) feet together standing on a hemisphere ball; (5) tandem standing on a hemisphere ball. Pairwise comparison to establish the progression: ^1^ Significant differences with kneeling on a foam mat. ^2^ Significant differences with feet together standing on the floor. ^3^ Significant differences with tandem standing on the floor.

The repeated measure ANOVA showed significant differences in the within-subject factor *exercise variations* (F = 65.701; *p* < 0.001; *f* = 2.54) but not in the between-subject factor *sex* (F = 0.579; *p* = 0.464; *f* = 2.56). However, the variations 4 and 5 (the most difficult) showed between-sex effect size differences of *d* = 0.88 and *d =* 0.68 (large-medium), respectively (Figure 3). Figure 4 shows the pairwise comparisons in mean lumbopelvic acceleration between the exercise variations and the resulting intensity progression based on the difficulty the participants had in maintaining each Pallof press position. The Pallof press variations performed in standing position on the hemisphere ball (feet together standing: 0.55 m/s^2^; tandem stance: 0.61 m/s^2^) showed higher mean acceleration values than those variations performed in standing position on the floor (feet together standing: 0.22 m/s^2^; tandem stance: 0.31 m/s^2^) and kneeling on the foam pad (0.17 m/s^2^). In addition, a tandem stance on the floor produced higher lumbopelvic accelerations than kneeling on the foam pad.

## 4. Discussion

Nowadays, different Pallof press variations are performed in sports and fitness settings varying the body position, using a cable or elastic band grip, carrying out limb movements, changing the support surface, etc. [22,23,24,25,26]. However, despite the popularity of this exercise, the intensity/difficulty of these Pallof press variations is unknown, and therefore, it is difficult to control the training load and establish exercise progressions. To the best of our knowledge, this is the first study that provides objective data from a smartphone accelerometer that allows us to compare and rank the postural control challenge imposed by five different isometric variations of the Pallof press exercise performed in different body positions and on different support surfaces (Figure 3). The main findings were: (i) the good data consistency; (ii) the absence of sex differences in postural control during the exercise variations; and (iii) that the Pallof press performance in standing position rather than kneeling, and on a hemisphere ball compared to more stable surfaces, increased the exercise difficulty.

As previously mentioned, the data consistency was good (ICC ≥ 0.72, typical errors ≤ 17%) and slightly better than those obtained in previous smartphone accelerometer studies on other CS exercises [11,12]. Regarding the between-sex analyses, as was expected considering the small sample size, males and females did not show significant differences in postural control when performing the Pallof press variations. However, the medium to large effect size (0.68 < *d* < 0.88) differences found between females and males in the two most difficult variations (feet together standing on a hemisphere ball and tandem standing on a hemisphere ball) suggest that sex effect cannot be rejected (Figure 3). Females’ better postural control could be related to anthropometric differences compared to males. The greater mass of men implies a higher mass supported on the hemisphere ball [11] and the greater height of men results in a higher height of the center of gravity and the lateral forces applied by the elastic band, which increases the tilting moment on the participant’s body. Both circumstances pose a greater challenge and could hinder men’s postural control in these variations. These results seem to be in line with previous findings in a posturographic study on conventional floor-based CS exercises [11], which found better postural control in females than in males in most exercises (i.e., lower lumbopelvic accelerations during bridging and bird-dog exercises). However, comparing the results of both studies is difficult, since the exercises are performed in different positions (i.e., lying or quadruped positions vs. standing or kneeling positions), and the level of the postural control is affected by variables that were not controlled in either study (i.e., training experience with these exercises, participants’ morphological characteristics, etc.), and the sample size of the current study is smaller. Therefore, further research is needed to understand the role of sex in the performance of the Pallof press and other CS exercises.

As Figure 4 shows, one of the main findings of the study was that, in general, the standing variations imposed higher postural demands than the kneeling variation. This could be probably caused by (i) the smaller base of support and the higher center of gravity while standing, which reduces the fall angle of the body; and (ii) the higher height of the lateral force applied by the elastic band, which increases the tilting moment on the participant’s body. On the other hand, despite the lower lateral base of support when the feet were placed in tandem position compared to the feet together position, no significant lumbopelvic acceleration differences were observed between these feet positions in standing, nor in the variations performed on the floor or in those performed on the hemisphere ball. However, as shown in the images presented in Figure 1, although in each standing variation (especially the variation on the hemisphere ball) the participants leaned laterally in the opposite direction to the lateral force applied by the elastic band to maintain the position, this strategy was mainly observed during the variations performed with the feet in tandem position (i.e., with lower lateral base of support). Further research is needed to understand the postural and neuromuscular effect of these and other foot positions on the Pallof press execution better. Finally, regarding the comparison between the Pallof press variations performed on the hemisphere ball and those performed on the floor (Figure 3), current results support the use of unstable surfaces (i.e., hemisphere balls, Swiss balls, suspension bands, and others) as criteria to increase the intensity of the CS exercises [11,12,32].

The findings of this study are limited to physically active individuals, therefore further research is needed to determine CS exercise progressions in different populations (athletes with different stability demands, patients with low back pain or balance deficits, etc.). Moreover, although the conventional Pallof press execution implies elbow flexion-extension movements, the Pallof press variations analyzed in this study were performed with the elbows held in full extension (i.e., with the largest lever arm). This was conducted to avoid sudden upper limb movements which could have altered the lumbopelvic acceleration recording and affected the data reliability. Finally, as previously mentioned, the small sample size limited the significant differences between sexes in some of the Pallof press variations. Future studies should explore the conventional Pallof press using other positions such as seated, half-kneeling, lunge, ¼ squat, etc., in a large sample of males and females.

## 5. Conclusions

Based on the current results, the Pallof press performance in standing rather than kneeling, and on a hemisphere ball compared to more stable surfaces, increased the exercise difficulty in physically active young males and females. Overall, this information can be used by clinicians, physical trainers, and researchers to modulate the difficulty and establish progressions for the Pallof press exercise. In this sense, standing Pallof press variations seem to be a better option than kneeling variations (e.g., for both, athletes and people who perform physical activity for health and well-being) to increase exercise difficulty while performing tasks in more functional positions, as well as to reduce stress on the knees (e.g., in individuals with knee injuries). On the other hand, kneeling positions may be more appropriate to reduce lower limb involvement and exercise difficulty, allowing the increase of the magnitude of the lateral force applied on the participant (i.e., using stiffer elastic bands or increasing the resistance mobilized on cable-pulley machines) and thus the activation of the core muscles. Regarding the use of the hemisphere ball, performing Pallof press variations on this unstable surface represents a significant challenge for many participants, so they seem to be indicated for people with high postural control. In this sense, the great difficulty in maintaining body posture on the hemisphere ball during this exercise could reduce the ability of less experienced participants to focus on the core structures, transforming the Pallof press into a whole-body balance exercise rather than a CS exercise.

## Figures and Tables

**Table 1:** Descriptive statistics and absolute and relative reliability.

	Variables	Session 1 (Mean ± SD)	Session 2 (Mean ± SD)	Change in Mean (95% CL)	Typical Error (%) (95% CL)	ICC_(3,1)_ (95% CL)
ISOMETRIC PALLOF-PRESS (m/s^2^)	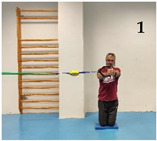	0.17 ± 0.05	0.17 ± 0.06	−0.01 (−0.03–0.02)	0.03 (17%) (0.02–0.05)	0.74 (0.31–0.92)
	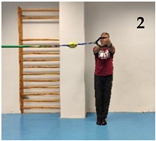	0.25 ± 0.07	0.22 ± 0.06	−0.04 (−0.07–−0.01)	0.03 (14%) (0.02–0.06)	0.80 (0.42–0.94)
	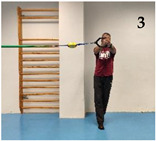	0.29 ± 0.09	0.31 ± 0.10	0.01 (−0.03–0.05)	0.04 (15%) (0.03–0.07)	0.81 (0.46–0.94)
	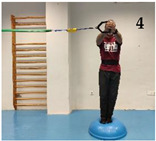	0.57 ± 0.17	0.55 ± 0.15	0.00 (−0.09–0.08)	0.09 (16%) (0.06–0.16)	0.72 (0.25–0.91)
	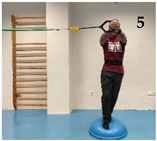	0.59 ± 0.12	0.61 ± 0.21	0.07 (−0.03–0.17)	0.09 (14%) (0.06–0.17)	0.82 (0.32–0.95)

SD: standard deviation; CL: confidence limits; ICC: intraclass correlation coefficient. Pallof press variations: (1) kneeling on a foam pad; (2) feet together standing on the floor; (3) tandem stance on the floor; (4) feet together standing on a hemisphere ball; (5) tandem stance on a hemisphere ball.

## Data Availability

The datasets obtained and analyzed for the current research can be provided by the authors of this study.
